# Elevated free cholesterol levels due to impaired reverse cholesterol transport are a risk factor for polymicrobial sepsis in mice

**DOI:** 10.1016/j.jbc.2024.107974

**Published:** 2024-11-05

**Authors:** Qian Wang, Ling Guo, Dan Hao, Misa Ito, Chieko Mineo, Philip W. Shaul, Xiang-An Li

**Affiliations:** 1 Saha Cardiovascular Research Center, University of Kentucky, Lexington, Kentucky, USA; 2 Department of Pediatrics, University of Texas Southwestern Medical Center, Dallas, Texas, USA; 3 Lexington VA Healthcare System, Lexington, Kentucky, USA; 4 Department of Physiology, University of Kentucky, Lexington, Kentucky, USA

**Keywords:** SR-BI, Scarb1, cholesterol, reverse cholesterol transport, hemolysis, sepsis

## Abstract

Dysregulated lipid metabolism is commonly observed in septic patients, but how it contributes to sepsis remains largely unknown. Reverse cholesterol transport (RCT) is crucial for regulating cholesterol metabolism in circulation. During RCT, high-density lipoprotein collects cholesterol from peripheral tissues and transports it to the liver’s scavenger receptor BI (SR-BI), where SR-BI mediates the uptake of cholesteryl esters (CEs) from high-density lipoprotein for excretion *via* bile. In this study, we utilized AlbCreSR-BI^fl/fl^ mice, a model with impaired RCT, to investigate the impact of RCT on sepsis. We found that AlbCreSR-BI^fl/fl^ mice were significantly more susceptible to cecal ligation and puncture (CLP)-induced polymicrobial sepsis, with a survival rate of 14.3% compared to 80% in SR-BI^fl/fl^ littermates. Mechanistically, sepsis disrupted cholesterol metabolism, causing a 4.8-fold increase in free cholesterol (FC) levels and a 4-fold increase in the FC/CE ratio in AlbCreSR-BI^fl/fl^ mice compared to SR-BI^fl/fl^ littermates. This disruption led to hemolysis and death. Notably, administering the cholesterol-lowering drug probucol normalized FC levels and the FC/CE ratio, and significantly improved survival in CLP-AlbCreSR-BI^fl/fl^ mice. However, probucol treatment reduced survival in CLP-low-density lipoprotein receptor knockout mice, which had elevated CE levels with a low FC/CE ratio. These results highlight that elevated FC levels with high FC/CE ratio are a risk factor for sepsis. Therefore, selectively targeting elevated FC levels and FC/CE ratio could be a promising therapeutic strategy for managing sepsis.

Sepsis is a life-threatening organ dysfunction caused by dysregulated host response to infection (1). While extensive efforts have been made to assess the dysregulated inflammatory response, targeting one or another component of the inflammatory pathways has had little impact on patient survival (2). Better understanding of the dysregulated host response, especially dysregulated metabolism in sepsis, may provide novel insights for sepsis.

Dysregulated lipid metabolism is observed in sepsis (3), but how it contributes to sepsis remains poorly understood. Low HDL is a well-documented phenotype in septic patients. Clinical studies showed that high-density lipoprotein (HDL)-cholesterol abundance is markedly reduced in septic patients and correlates with a poor prognosis (4, 5, 6, 7); animal studies showed low HDL is a risk factor for sepsis (6, 8). A major function of HDL is to regulate cholesterol metabolism through reverse cholesterol transport (RCT), in which HDL transports peripheral cholesterol to the liver, where scavenger receptor BI (SR-BI or Scarb1) mediates the uptake of cholesteryl esters from HDL for excretion *via* bile. While it is well-established that HDL-SR-BI–mediated RCT plays a critical role in protection against cardiovascular diseases (9, 10, 11), whether HDL-SR-BI–mediated RCT contributes to sepsis remains to be determined. Addressing this issue may provide a therapeutic target for sepsis.

As an HDL receptor, SR-BI is highly expressed in liver and steroidogenic tissues (12, 13). Using SR-BI KO mice, we reported that SR-BI protects against sepsis (14, 15). Later studies showed that SR-BI protects against sepsis through multiple mechanisms, including prevention of nitro oxide-induced cytotoxicity (14), promotion of LPS clearance (15, 16), suppression of inflammatory signaling in macrophages (15, 17), and mediating induced glucocorticoid GC production in adrenal gland (16, 18, 19, 20, 21). Using mutant SR-BI mice that have a 90% decrease in SR-BI expression in the liver, we previously reported that liver SR-BI protects against cecal ligation and puncture (CLP)-induced sepsis (22). However, using hypo-AlbCreSR-BI^fl/fl^ mice, Huby’s group found no difference in survival between CLP-challenged hypo-AlbCreSR-BI^fl/fl^ and hypo-SR-BI^fl/fl^ mice (19). Thus, the role of hepatic SR-BI in sepsis needs to be verified.

In this study, we generated new AlbCreSR-BI^fl/fl^ mice (liver-specific SR-BI KO mice) as an impaired RCT model to assess the impact of RCT on sepsis and to clarify the role of hepatic SR-BI. The SR-BI^fl/fl^ mice had normal SR-BI expression in the liver and the AlbCreSR-BI^fl/fl^ mice had complete depletion in SR-BI expression in the liver. We found that sepsis induces a profound increase in plasma free cholesterol (FC) levels and the FC/ cholesteryl ester (CE) ratio in AlbCreSR-BI^fl/fl^ mice due to impaired RCT. We showed that probucol, a cholesterol-lowering drug, normalizes FC levels and the FC/CE ratio and rescues AlbCreSR-BI^fl/fl^ mice. Our findings suggest that elevated FC levels with high FC/CE ratio are a risk factor for sepsis and selectively targeting dysregulated cholesterol metabolism may be an effective therapeutic approach for sepsis.

## Results

### Generation of liver-specific SR-BI KO mice

**Figure 1:**
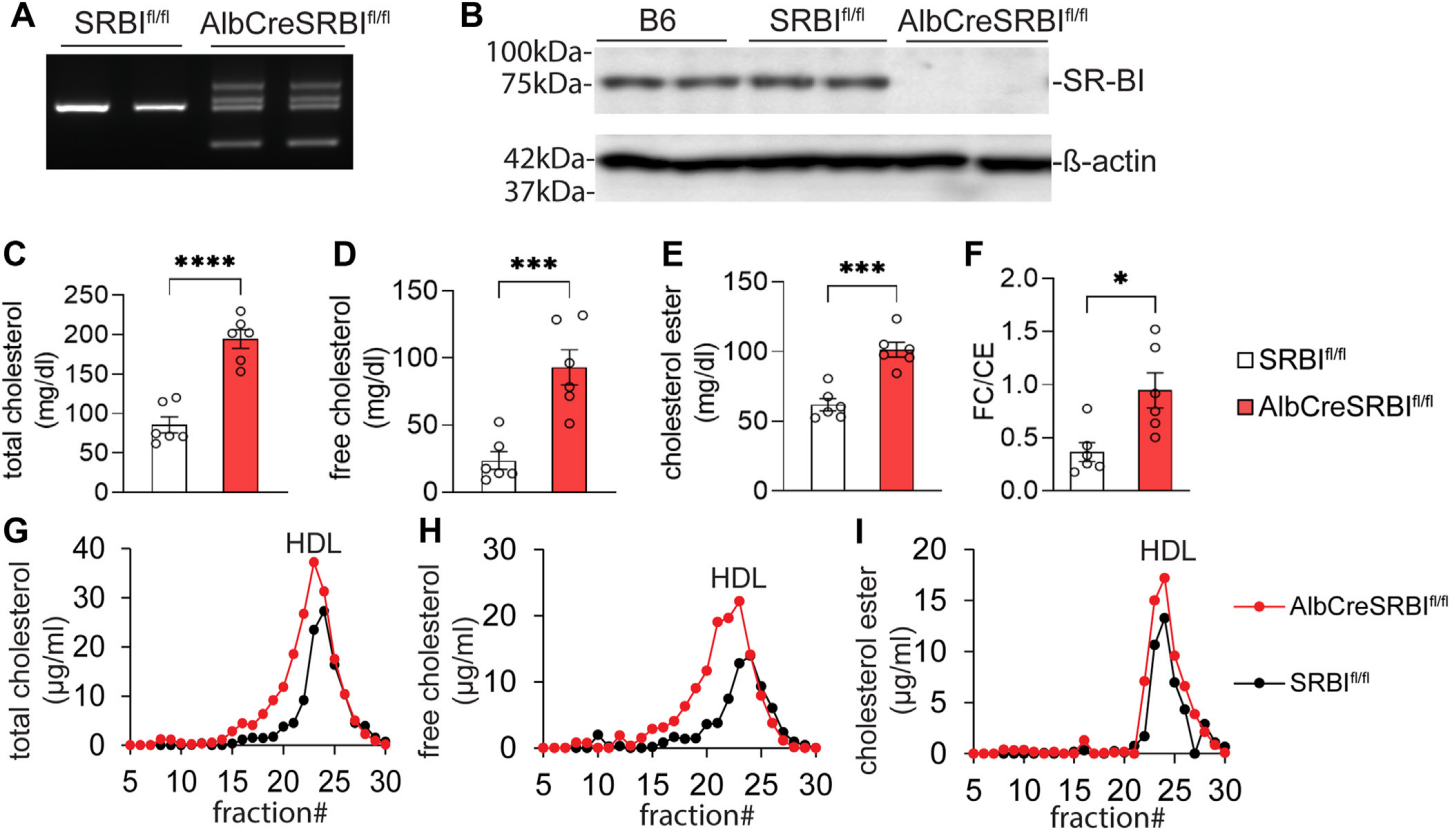
**Generation of liver-specific SR-BI KO mice.***A*, PCR genotyping. *B*, Western blot of SR-BI expression in the liver. Serum levels of total cholesterol (C), free cholesterol (D), cholesteryl ester (E), and free cholesterol/cholesteryl ester ratio (F) were analyzed in AlbCreSR-BI^fl/fl^ and SR-BI^fl/fl^ mice. n = 6 for each group, mean ± SEM. Data were analyzed by student *t* test. ∗*p* < 0.05, ∗∗∗*p* < 0.001, and ∗∗∗∗*p* < 0.0001. *G-I*, representative lipoprotein profile. Total cholesterol (G), free cholesterol (H), and cholesteryl ester (I). n = 2 to 3 for each group. SR-BI, scavenger receptor BI.

We previously generated SR-BI^fl/fl^ mice (11). We bred SR-BI^fl/fl^ and AlbCre mice to generate AlbCreSR-BI^fl/fl^ mice (Fig. 1*A*). Western blotting analysis showed complete deletion of SR-BI expression in the liver of AlbCreSR-BI^fl/fl^ mice. SR-BI expression in the liver of SRBI^fl/fl^ mice was similar to that of C57BL/6J (B6) mice (Fig. 1*B*). Deficiency in hepatic SR-BI impaired cholesterol metabolism, as shown by significant increases in total cholesterol (TC), FC, CE levels, and FC/CE ratio in AlbCreSR-BI^fl/fl^ *versus* SRBI^fl/fl^ mice (Fig. 1, *C*–*E*). Lipoprotein profile analysis showed that cholesterol increased mostly on HDL fraction in AlbCreSR-BI^fl/fl^ mice (Fig. 1, *G*–*I*).

### AlbCreSR-BI^fl/fl^ mice are susceptible to polymicrobial sepsis

**Figure 2:**
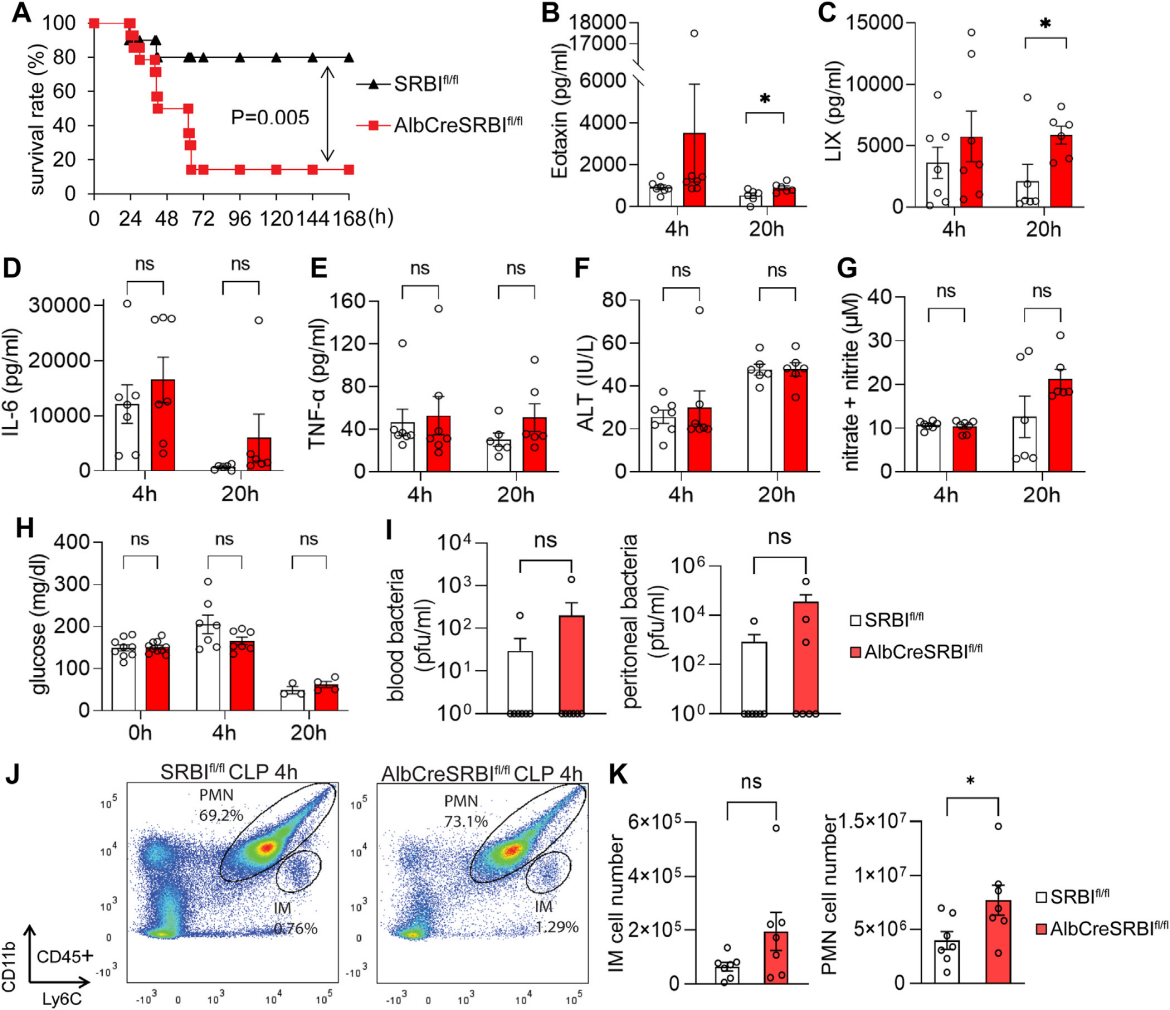
**AlbCreSR-BI**^**fl/fl**^**mice are more susceptible to polymicrobial sepsis.** AlbCreSR-BI^fl/fl^ and SR-BI^fl/fl^ mice were treated with CLP (23G needle, half ligation). *A*, survival analysis. Survival was monitored for 7 days. The data were expressed as the percentage of mice surviving at indicated times, and survival was analyzed by the log-rank test. n = 10 to 14. *B-E*, serum cytokine. *F*, ALT levels. *G*, nitrate and nitrite levels. *H*, glucose levels at 0 h, 4 h, and 20 h post-CLP. Student’s *t* test was used to compare the 2 groups at the indicated time points. n = 6 to 7, mean ± SEM. ∗*p* < 0.05 *versus* SR-BI^fl/fl^ mice. *I*, blood and peritoneal fluid were analyzed for bacteria load at 20 h post-CLP. *J* and *K*, peritoneal fluid was collected at 4 h post-CLP. Peritoneal neutrophils (PMN, CD11b^hi^ Ly6C^hi^) and inflammatory monocytes (IM, CD11b^int^ Ly6C^hi^) were gated by CD11b and Ly6C expression on CD45^+^ cells. Student’s *t* test was used to compare the 2 groups. n = 6 to 7 for each group, mean ± SEM. ∗*p* < 0.05 *versus* SR-BI^fl/fl^ mice. ALT, alanine transaminase; CLP, cecal ligation and puncture; SR-BI, scavenger receptor BI.

We induced sepsis with CLP to investigate the impact of impaired RCT on sepsis. We observed a significant decrease in survival in AlbCreSR-BI^fl/fl^ mice, indicated by a survival rate of only 14.3% in AlbCreSR-BI^fl/fl^ mice compared to 80% in SR-BI^fl/fl^ littermates (Fig. 2*A*). To understand why AlbCreSR-BI^fl/fl^ mice are susceptible to polymicrobial sepsis, we assessed immune response by measuring 31 plasma cytokines/chemokines at 4 h and 20 h after CLP. No significant differences in cytokines were observed at 4 h post-CLP and only a few cytokines were moderately increased at 20 h post-CLP in AlbCreSR-BI^fl/fl^ mice compared to SR-BI^fl/fl^ littermates (Fig. 2, *B*–*E*, and Supporting information Table S1). We examined alanine transaminase (ALT) levels as an indicator of liver injury. While there was a significant increase in ALT levels in both SR-BI^fl/fl^ and AlbCreSR-BI^fl/fl^ mice at 20 h post-CLP compared to 4 h post-CLP, no difference in ALT levels was observed between SR-BI^fl/fl^ and AlbCreSR-BI^fl/fl^ mice at either 4- or 20-h post-CLP. Thus, liver injury may not account for the significant increase in mortality rate in AlbCreSR-BI^fl/fl^ mice (Fig. 2*F*). We quantified NOx (nitrate and nitrite) and glucose concentrations but did not find differences between SR-BI^fl/fl^ and AlbCreSR-BI^fl/fl^ mice (Fig. 2, *G* and *H*). We then examined the bacteria loads in blood and peritoneal and found no differences (Fig. 2*I*). The flow cytometry analysis showed a higher number of peritoneal neutrophils in AlbCreSR-BI^fl/fl^ mice than SR-BI^fl/fl^ mice (Fig. 2, *J* and *K*). The moderately changed inflammatory cytokines and immune cell recruitments may not justify the significant increase in mortality rate in AlbCreSR-BI^fl/fl^ mice.

### AlbCreSR-BI^fl/fl^ mice have elevated FC levels and FC/CE ratio, which lead to hemolysis in sepsis

**Figure 3:**
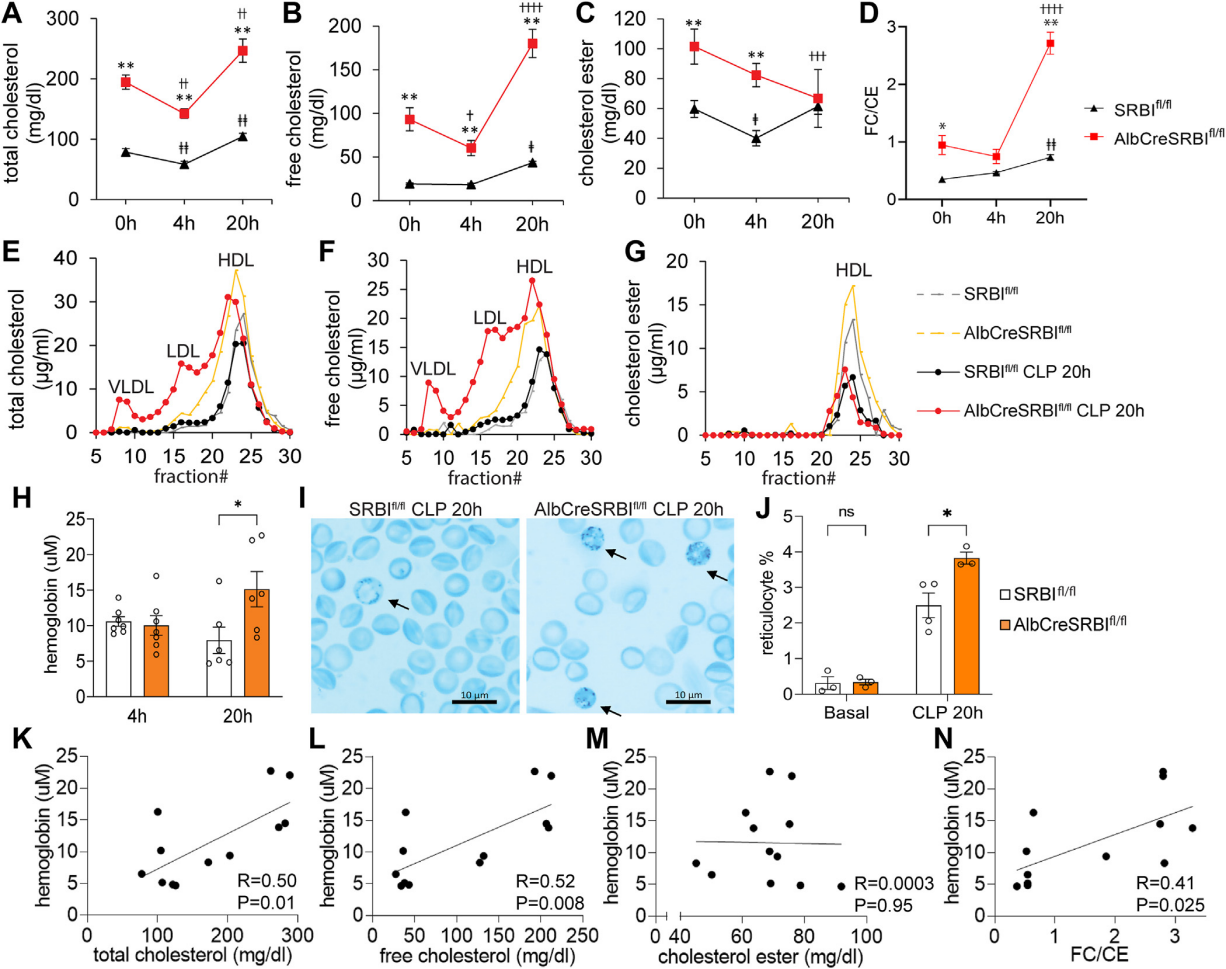
**Elevation in plasma free cholesterol levels and hemolysis in CLP-challenged AlbCreSR-BI**^**fl/fl**^**mice.** AlbCreSR-BI^fl/fl^ and SR-BI^fl/fl^ mice were treated with CLP (23G needle, half ligation). Serum levels of total cholesterol (A), free cholesterol (B), cholesteryl ester (C), and FC/CE ratio (D) were analyzed at 0 h, 4 h and 20 h after CLP. Data were analyzed by two-way ANOVA followed by post hoc analysis using Tukey’s test, n = 6 to 7 for each group, mean ± SEM. *E-G*, representative lipoprotein profile (E, total cholesterol; F, free cholesterol; and G, cholesteryl ester levels. n = 2–3 for each group). *H*, serum hemoglobin levels were analyzed at 4 h and 20 h after CLP. Student’s *t* test was used to compare the 2 groups, n = 6 to 7. *I*, peripheral blood reticulocyte staining from AlbCreSR-BI^fl/fl^ and SR-BI^fl/fl^ mice at CLP 20 h. *J*, quantification of reticulocytes before CLP and at CLP 20h. Student’s *t* test was used to compare the 2 groups, n = 3 to 4. Correlation analysis between cholesterol levels (K, total cholesterol, L, free cholesterol, M, cholesteryl ester, N, FC/CE ratio) and hemoglobin levels at CLP 20 h, using Pearson’s correlation test. n = 12. ∗*p* < 0.05 and ∗∗*p* < 0.01, AlbCreSR-BI^fl/fl^*versus* SR-BI^fl/fl^ mice. ⴕp < 0.05, ⴕⴕ*p* < 0.01, ⴕⴕⴕ*p* < 0.001, and ⴕⴕⴕⴕ*p* < 0.0001, septic AlbCreSR-BI^fl/fl^ mice *versus* nonseptic AlbCreSR-BI^fl/fl^ mice. ⱡ*p* < 0.05 and ⱡⱡ*p* < 0.01 septic SR-BI^fl/fl^ mice *versus* nonseptic SR-BI^fl/fl^ mice. CLP, cecal ligation and puncture; SR-BI, scavenger receptor BI

While a dysregulated immune response remains a significant contributor to sepsis, extensive clinical trials aimed at modulating this response have yielded disappointing results. These unsuccessful trials prompted the sepsis community to explore novel target beyond immune response (23, 24). To further understand why AlbCreSR-BI^fl/fl^ mice are susceptible to polymicrobial sepsis, we looked at the cholesterol metabolism, given the prominent role of hepatic SR-BI in regulating cholesterol metabolism (13). We first looked at how sepsis alters cholesterol metabolism. Compared to nonseptic SR-BI^fl/fl^ mice, CLP-SR-BI^fl/fl^ mice had a moderate increase in FC levels and FC/CE ratio, and no changes in CE levels; in contrast, compared to nonseptic AlbCreSR-BI^fl/fl^ mice, CLP-AlbCreSR-BI^fl/fl^ mice had a 2-fold increase in FC levels, a 30% decrease in CE levels, and a 3-fold increase in FC/CE ratio (Fig. 3, *A*–*D*). We then looked at how impaired RCT affects cholesterol metabolism under sepsis conditions. Compared to CLP-SR-BI^fl/fl^ mice, CLP-AlbCreSR-BI^fl/fl^ mice had a 4.8-fold increase in FC levels, no changes in CE levels, and a 4-fold increase in FC/CE ratio at 20 h post-CLP (Fig. 3, *A*–*D*). These indicate that impaired RCT have profound effect on cholesterol metabolism in sepsis, leading to marked increases in FC levels and in FC/CE ratio. Lipoprotein profiling analysis showed that the most FC appear in HDL and LDL factions, while CE appears in HDL fractions 20 h post-CLP (Fig. 3, *E*–*G*).

FC is located on the surface of lipoproteins, while CE is situated in the inner core. An elevation in FC levels and FC/CE ratio results in more FC molecules on the surface of lipoproteins. Early *in vitro* studies demonstrated that FC in lipoproteins can freely exchange cholesterol with red blood cells (RBCs). This exchange is bidirectional and driven by a concentration gradient. High levels of FC in lipoproteins transfer FC to the RBC membrane, making RBCs more susceptible to hemolysis (25, 26, 27, 28). Hemolysis is an independent risk factor for sepsis (29), due to organ injury caused by highly toxic hemoglobin and hemin form broken RBCs. Thus, we looked at hemolysis by measuring extracellular hemoglobin and reticulocytes in circulation. We observed a 2-fold increase in hemoglobin concentrations (Fig. 3*H*) and a significant increase in reticulocytes (Fig. 3, *I* and *J*) in AlbCreSR-BI^fl/fl^ mice compared to SR-BI^fl/fl^ mice at 20 h post-CLP challenge. Interestingly, hemoglobin levels were positively correlated with FC levels and the FC/CE ratio, but not with CE levels (Fig. 3, *K*–*N*), supporting the role of FC in inducing hemolysis.

### AlbCreSR-BI^fl/fl^ mice feature upregulated cholesterol biosynthesis and downregulated cholesterol secretion in sepsis

**Figure 4:**
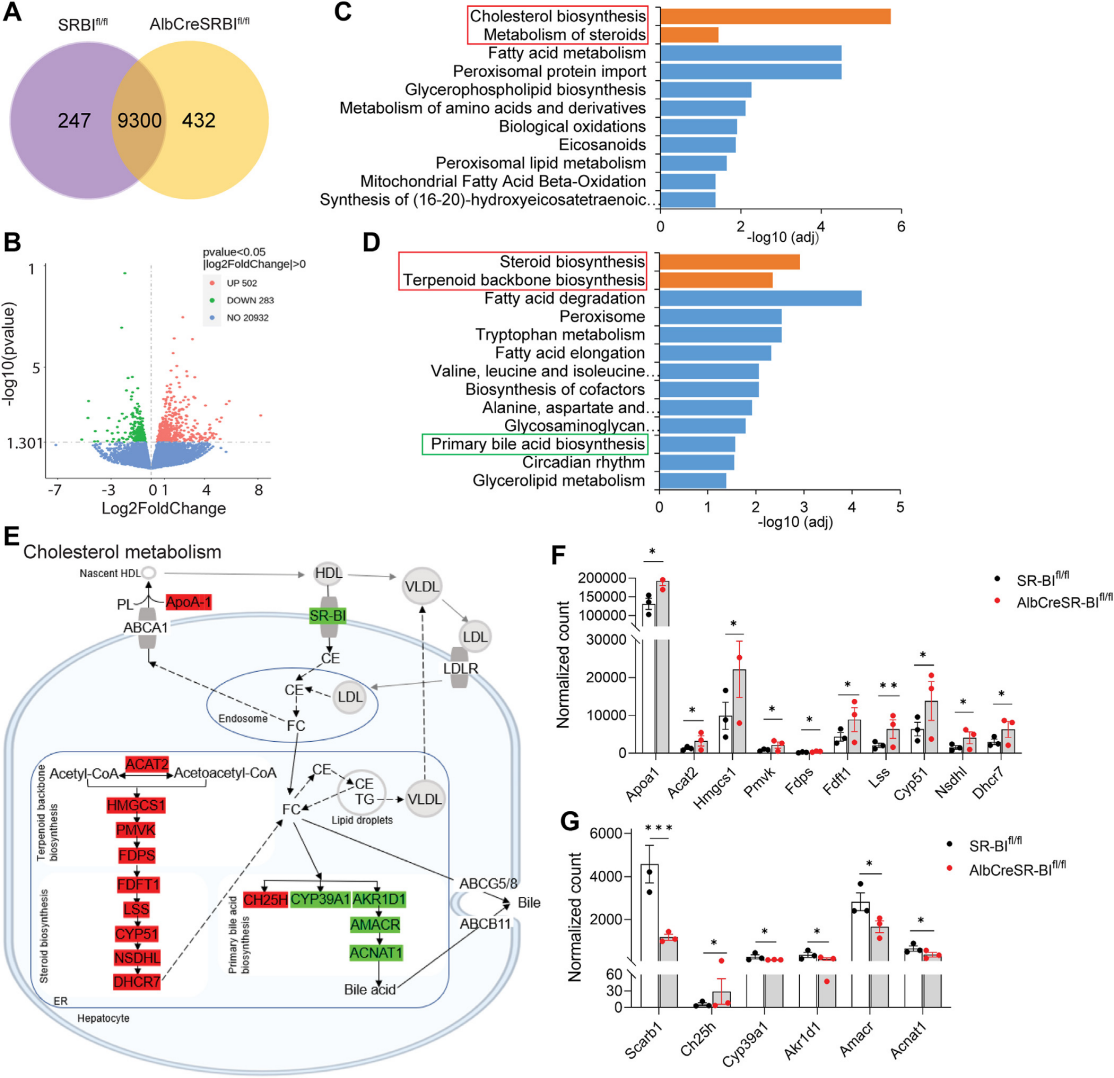
**AlbCreSR-BI**^**fl/fl**^**mice feature upregulated cholesterol biosynthesis and downregulated cholesterol secretion in sepsis.** Liver RNA-seq analysis was performed on AlbCreSR-BI^fl/fl^ and SR-BI^fl/fl^ mice at 20 h post-CLP (n = 3). *A*, Venn diagram shows the distribution of overlapping genes and unique DEGs. *B*, volcano plot shows upregulated (*red*) and downregulated (*green*) genes. *C*, top enriched reactome for upregulated (*orange bar*) and downregulated DEGs (*blue bar*), padj < 0.05. *D*, top enriched KEGG pathways for upregulated (*orange bar*) and for downregulated DEGs (*blue bar*), padj < 0.05. *E*, modified cholesterol metabolism pathway from KEGG. *F*, normalized count of DEGs of steroid biosynthesis. *G*, normalized count of DEGs of bile acid biosynthesis. ALT, alanine transaminase; CLP, cecal ligation and puncture; DEG, differentially expressed gene; KEGG, Kyoto encyclopedia of genes and genomes; SR-BI, scavenger receptor BI.

To understand why AlbCreSR-BI^fl/fl^ mice have elevated FC, we conducted RNA-seq analysis 20 h post-CLP. 247 and 432 distinctive genes were detected in SR-BI^fl/fl^ and AlbCreSR-BI^fl/fl^ mice (Fig. 4*A*). Compared to SR-BI^fl/fl^ mice, 502 upregulated and 283 downregulated differentially expressed genes were identified in AlbCreSR-BI^fl/fl^ mice (Fig. 4*B*). Reactome and Kyoto encyclopedia of genes and genomes pathway enrichment analyses identified 2 significantly enriched pathways: cholesterol biosynthesis and steroid biosynthesis (Fig. 4, *C*–*F*). Kyoto encyclopedia of genes and genomes pathway enrichment analysis also revealed downregulated differentially expressed genes enriched in primary bile acid biosynthesis (Fig. 4, *D* and *E* and *G*). In addition, an early report showed that a deficiency in SR-BI caused a 90% decrease in lecithin-cholesterol acyltransferase activity (30), an enzyme crucial for converting FC into CE on HDL. This also disrupts FC metabolism, leading to high FC levels and FC/CE ratio.

Taken together, the increase in cholesterol biosynthesis, decrease in bile acid biosynthesis, and a lack of lecithin-cholesterol acyltransferase activity are likely responsible for elevated FC levels and FC/CE ratio in AlbCreSR-BI^fl/fl^ mice in sepsis.

### Normalization of FC levels and FC/CE ratio by probucol rescues septic AlbCreSR-BI^fl/fl^ mice

**Figure 5:**
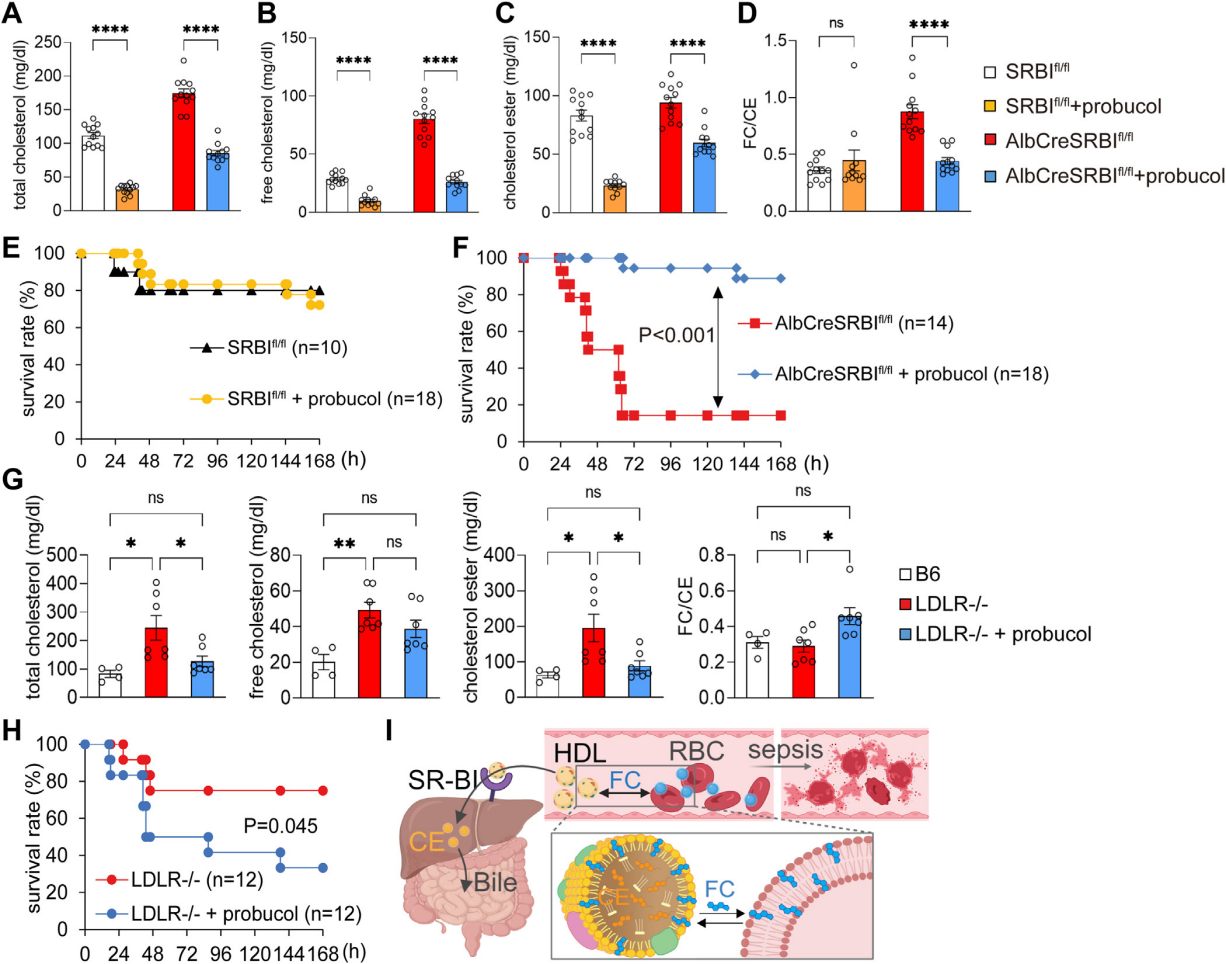
**Probucol normalizes free cholesterol levels and rescues AlbCreSR-BI**^**fl/fl**^**mice.** AlbCreSR-BI^fl/fl^ and SR-BI^fl/fl^ mice were pretreated with probucol-containing diet for 3 days and plasma cholesterol levels were quantified. *A*, total cholesterol. *B*, free cholesterol. *C*, cholesteryl ester. *D*, FC/CE ratio. Student’s *t* test was used to compare the 2 groups. n = 12 for each group, mean ± SEM. Then, the probucol-treated SR-BI^fl/fl^ (E) and AlbCreSR-BI^fl/fl^ (F) mice were challenged with CLP. The survival was compared to mice without probucol treatment (as described in Fig. 2*A*) and analyzed using the log-rank test. LDLR^−/−^ mice were pretreated with probucol-containing diet for 3 days. Plasma total cholesterol, free cholesterol, and cholesteryl ester were quantified (G). Student’s *t* test was used to compare the 2 groups. n = 7 for each group, mean ± SEM. Then, the probucol-treated LDLR^−/−^ mice were challenged with CLP. The survival was analyzed using the log-rank test with mice without probucol treatment as control (H). ∗*p* < 0.05, ∗∗*p* < 0.01, ∗∗∗*p* < 0.001, ∗∗∗∗*p* < 0.0001. *I*, schematic model of impaired reverse cholesterol transportation contributes to sepsis. SR-BI facilitates the uptake of cholesteryl ester (CE) from high-density lipoprotein (HDL) and transports it to the liver for excretion *via* bile. This process is known as reverse cholesterol transport (RCT). A deficiency of hepatic SR-BI leads to impaired RCT. The impaired RCT results in high free cholesterol (FC) and FC/CE ratio in lipoproteins. The FC molecules are located on the surface of lipoproteins. They freely diffuse to red blood cells (RBCs), which renders RBCs susceptible to rupture, resulting in hemolysis and septic death. Created with Biorender.com. CLP, cecal ligation and puncture; SR-BI, scavenger receptor BI.

Finally, to determine a causal relationship between elevated FC levels with high FC/CE ratio and septic death, we normalized FC levels and the FC/CE ratio with probucol. Probucol is a cholesterol-lowering drug. It inhibits cholesterol synthesis and suppresses cholesterol efflux from peripheral tissues through ABCA1 (31, 32, 33). Thus, probucol is a useful drug to normalize the elevated FC levels in AlbCreSR-BI^fl/fl^ mice. As shown in Fig. 5, *A*–*D*, upon probucol treatment, the TC, FC, CE levels, and the FC/CE ratio in AlbCreSR-BI^fl/fl^ mice were normalized to the levels in SR-BI^fl/fl^ mice. Importantly, probucol treatment significantly rescued CLP-AlbCreSR-BI^fl/fl^ mice, increasing the survival rate from 14.3% to 88.9% (Fig. 5*F*). Probucol treatment did not affect survival of CLP-SR-BI^fl/fl^ mice (Fig. 5*E*). Thus, probucol improves survival in mice with elevated FC levels.

Probucol also decreased TC and CE in AlbCreSR-BI^fl/fl^ mice, suggesting an alternative explanation for its protective effects. To investigate further, we used another high cholesterol mouse model, low-density lipoprotein receptor knockout (LDLR^−/−^) mice. These mice exhibited hypercholesterolemia with TC levels similar to AlbCreSR-BI^fl/fl^ mice. However, in LDLR^−/−^mice, cholesterol was predominantly in the form of CE, resulting in a low FC/CE ratio, similar to control C57BL/6J mice (Fig. 5*G*). This makes the LDLR^−/−^ mouse a useful model for addressing this concern. We treated the LDLR^−/−^ mice with probucol, which significantly reduced TC and CE levels but did not reduce FC levels or the FC/CE ratio (Fig. 5*G*). Interestingly, probucol-treated LDLR^−/−^ mice had significantly reduced survival compared to untreated LDLR^−/−^ mice upon CLP challenge (Fig. 5*H*). This contrasts with the increased survival observed in probucol-treated AlbCreSR-BI ^fl/fl^ mice (Fig. 5*F*). These findings demonstrate that cholesterol metabolism plays an important role in sepsis, with elevated FC levels and a high FC/CE ratio being risk factors, while high CE levels and a low FC/CE ratio are protective.

It is worth noting that probucol has activities beyond lowering cholesterol, such as anti-inflammatory and antioxidative stress effects. These activities may provide an alternative explanation for probucol’s protection in AlbCreSR-BI^fl/fl^ mice. If probucol protects against sepsis through these activities, it would be expected to protect against sepsis in SR-BI^fl/fl^, LDLR^−/−^, and AlbCreSR-BI^fl/fl^ mice. However, survival analysis showed that probucol treatment only benefits AlbCreSR-BI^fl/fl^ mice, has no effect on SR-BI^fl/fl^ mice, and actually harms LDLR^−/−^ mice. Thus, probucol provides protection by normalizing FC levels and the FC/CE ratio.

In sum, our study demonstrates that cholesterol metabolism contributes to sepsis (Fig. 5*I*). In AlbCreSR-BI^fl/fl^ mice, a deficiency in hepatic SR-BI–mediated reverse cholesterol transportation disrupts cholesterol metabolism, leading to marked increases in FC levels and a high FC/CE ratio, which causes hemolysis and septic death.

## Discussion

Dysregulated lipid metabolism is observed in sepsis (3), but how it contributes to sepsis remains poorly understood. In this study, using AlbCreSR-BI^fl/fl^ mice as an RCT-deficient model, we demonstrate that impaired RCT disputes cholesterol metabolism in sepsis, leading to elevated FC levels and an increased FC/CE ratio. The FC in lipoproteins freely diffuses into RBCs, making them susceptible to rupture, which results in hemolysis and septic death. Probucol treatment effectively normalizes FC levels and the FC/CE ratio, rescuing septic AlbCreSR-BI^fl/fl^ mice. Our study suggests that elevated plasma FC levels with a high FC/CE ratio are risk factors for sepsis, and probucol treatment may serve as an effective therapy for septic patients with these conditions.

### Clarification of role of hepatic SR-BI in protection against sepsis

We previously reported that hepatic SR-BI protects against CLP-induced sepsis using Scarb1^I179N^ mice. These mice have a mutant I179N, resulting in 90% depletion of hepatic SR-BI (22). However, using a hypo-AlbCreSR-BI^fl/fl^ mouse model, Huby’s group showed that the hypo-AlbCreSR-BI^fl/fl^ mice are not more susceptible to CLP-induced sepsis, compared to hypo-SR-BI^fl/fl^ mice (19). An issue with the hypo-AlbCreSR-BI^fl/fl^ mouse model is that the floxed SR-BI mice are hypomorphs (control SR-BI^fl/fl^ mice have a 90% whole-body depletion of SR-BI) due to the disruption of regulatory elements around exon 1 (34). In this study, we generated a new floxed SR-BI mouse by flanking exon 2, which had normal SR-BI expression in all tissues (11). We bred the floxed mice with AlbCre mice to generate AlbCreSR-BI^fl/fl^ mice and confirmed the protective role of hepatic SR-BI against polymicrobial sepsis.

### A precision medicine approach to target elevated FC may present an effective therapy for sepsis

Statins lower plasma cholesterol levels by inhibiting HMG-CoA reductase, the rate-limiting enzyme for *de novo* cholesterol synthesis (35). They also have anti-inflammatory and antioxidative effects. Due to these properties, statins have been tested for sepsis therapy, but results are controversial. Randomized trials show that atorvastatin prevents sepsis progression (36) but does not improve survival in patients with severe sepsis (37). A large cohort study found statins reduce sepsis incidence in cardiovascular patients (38). Another study showed statins lower sepsis risk in dialysis patients with chronic kidney disease (39). In a cohort study, 2.4% of statin-treated patients developed severe sepsis compared to 19% of nonstatin patients (40). High-potency statins are more effective in reducing sepsis mortality and complications (41). However, another study found no change in inflammatory response or coagulation with prior statin use (42) and advised caution (43). Meta-analyses indicate statins do not reduce sepsis mortality (44, 45, 46).

Our findings may provide an explanation for the controversial statin trials (36, 37, 38, 39, 44). Statins were used for all septic patients without distinguishing their cholesterol metabolism subtypes. Our findings suggest that lowering cholesterol without differentiating between FC and CE might not be beneficial and could even be harmful. We recommend monitoring FC levels and the FC/CE ratio in septic patients to determine if elevated FC levels with high FC/CE ratio are an endotype associated with poor outcomes. Emerging voices, including ours (20, 21, 47, 48), have called for an endotype-based precision medicine approach for sepsis therapy. We propose using a precision medicine approach to guide the use of probucol or statins, targeting septic patients with elevated FC levels and the FC/CE ratio.

## Experimental procedures

### Materials and methods

Materials are listed in Supporting information Table S2.

### Animals

We generated SR-BI^fl/fl^ mice by flanking exon 2 (11). The mice were backcrossed to C57BL/6J for 10 generations. We bred the floxed mice to AlbCre mice to generate AlbCreSR-BI^fl/fl^ mice (liver-specific SR-BI KO mice). Albcre mice in C57BL/6J background were bought from Jackson Lab (stock NO: 003574). Both male and female mice were used. Sex-matched SR-BI^fl/fl^ littermates were used as control. LDLR^−/−^ mice in C57BL/6J background were bought from Jackson Lab (stock NO: 002207). Mice were fed with a standard laboratory rodent diet. Animal experiments were approved by Animal Care and Use Committee of the University of Kentucky.

### CLP-induced sepsis model

CLP causes peritonitis, which leads to polymicrobial sepsis. We conducted CLP on 3-month-old mice as previously described (15). Mice were anesthetized and ligated at a half distance of the cecum and punched twice with a 23G needle. After surgery, 1 ml PBS was injected to resuscitate and survival was observed for 7 days. For probucol administration, mice were fed with a 0.5% probucol diet (probucol was dissolved in ethanol and sprayed on the normal rodent diet, and dried thoroughly) for 3 days before CLP.

### Biochemical assays

Mouse blood was collected from the abdominal artery at 4 h and 20 h following CLP, and the serum was stored at −80 °C for biochemical assays. Cytokines were analyzed by Eve Technologies using Mouse Cytokine/Chemokine Array 31-Plex. The cholesterol kit was from Wako. The hemoglobin kit was from Sigma (MAK115).

### Lipoprotein profiling

Fast protein liquid chromatography was used to separate lipoproteins as described (15).

### Analysis of leukocyte recruitment to peritoneum and bacteria load

Leukocyte recruitment to the peritoneum cavity was analyzed as described previously (8). Bacteria loads of blood and peritoneal fluid was analyzed as described previously (21).

### Reticulocyte counts

The tail blood was collected from mice and anticoagulated with EDTA. The collected blood was then mixed at a 1:1 ratio with a reticulocyte stain solution and incubated for 15 min at room temperature. Afterward, blood smears were prepared on microscope slides and evaluated under a light microscope. The reticulocyte count was determined by assessing the number of reticulocytes per 1000 erythrocytes, and this count was then converted into a percentage.

### RNA-seq analysis

The liver was collected from the mice at 20 h post-CLP. Total RNA was isolated by using RNeasy Kit. Sequencing and data analysis were performed by Novogene.

### Statistical analysis

The survival was analyzed by the Kaplan–Meier method with log-rank test. A two-tailed Student’s *t* test was used to compare the 2 groups. Comparing more than 2 groups was analyzed by one-way ANOVA. Pearson’s correlation test was applied to analyze correlation between 2 numerical data. Data were presented as means ± SEM. Significant differences were considered at *p* value <0.05. The statistical analysis was done using GraphPad Prism 9 (https://www.graphpad.com/).

## Data availability

All data are contained within the article.

## Supporting information

This article contains supporting information.

## Conflict of interest

The authors declare that they have no conflicts of interest with the contents of this article.
