# Supplementary material for: Elevated free cholesterol levels due to impaired reverse cholesterol transport are a risk factor for polymicrobial sepsis in mice
Source: J Biol Chem. 2024 Nov 5;300(12):107974. doi: 10.1016/j.jbc.2024.107974 (PMC11647492; doi:10.1016/j.jbc.2024.107974)
Supplement: Supporting Information [file mmc1.docx]

**Elevated free cholesterol levels due to impaired reverse cholesterol transport are a risk factor for polymicrobial sepsis in mice**

Qian Wang^1^, Ling Guo^1^, Dan Hao^1^, Misa Ito^1^, Chieko Mineo^2^, Philip W. Shaul^2^ and Xiang-An Li^1,3,4*^

**Supporting Information Table S1. List of cytokines that show no significant difference between AlbCreSR-BI^fl/fl^ and SR-BI^fl/fl^ mice treated with CLP.** AlbCreSR-BI^fl/fl^ and SR-BI^fl/fl^ mice were treated with CLP (23G needle, half ligation) for 4 and 20h. Serum was harvested and analyzed for cytokines levels. Data comparing AlbCreSR-BI^fl/fl^ and SR-BI^fl/fl^ mice were analyzed by student’s t-test. n = 6 – 7, mean ± SEM.

| List of cytokines (pg/ml) | | | | |  |  |
| --- | --- | --- | --- | --- | --- | --- |
|  | SRBI^fl/fl^ | | AlbCreSRBI^fl/fl^ | | P value | P value |
|  | CLP 4h | CLP 20h | CLP 4h | CLP 20h | CLP 4h | CLP 20h |
| Eotaxin | 928.12±114.8 | 528.5±123.73 | 3511.51±2334.17 | 906.52±90.42 | 0.31 | 0.04* |
| G-CSF | 34021.27±2612.96 | 33546.33±6754.84 | 32228.32±1701.55 | 37360.03±1934.56 | 0.58 | 0.61 |
| GM-CSF | 48.28±22.59 | 20.04±2.8 | 20.48±2.4 | 17.54±3.9 | 0.27 | 0.62 |
| IFNy | 48.54±39.97 | 7.46±2.66 | 3.85±0.98 | 7.83±1.53 | 0.31 | 0.91 |
| IL-1a | 667.26±96.88 | 471.27±87.1 | 651.87±85.87 | 521.16±36.71 | 0.91 | 0.61 |
| IL-1B | 40.06±30.64 | 9.4±3.61 | 45.98±35.67 | 13.54±1.4 | 0.90 | 0.32 |
| IL-2 | 42.81±23.36 | 14.51±3.29 | 9.14±1.6 | 14.48±4.42 | 0.20 | 1.00 |
| IL-3 | 11.67±7.31 | 3.44±1.7 | 2.31±0.49 | 1.99±0.57 | 0.25 | 0.45 |
| IL-4 | 3.59±2.02 | 1.22±0.67 | 2.25±1.44 | 1.13±0.45 | 0.60 | 0.91 |
| IL-5 | 402.58±74.35 | 33.76±12.78 | 559.27±106.08 | 33.31±10.17 | 0.25 | 0.98 |
| IL-6 | 12116.14±3503.55 | 668.62±192.79 | 16532.15±4132.36 | 6048.92±4260.83 | 0.43 | 0.26 |
| IL-7 | 28.42±11.57 | 7.17±1.52 | 8.18±1.67 | 15.11±9.55 | 0.13 | 0.45 |
| IL-9 | 127.47±25.34 | 161.01±34.38 | 104.19±18.1 | 120.99±13.55 | 0.47 | 0.32 |
| IL-10 | 604.92±170.32 | 325.81±152.05 | 459.42±124.86 | 245.72±186.83 | 0.51 | 0.75 |
| IL-12 (p40) | 100.28±35.32 | 52.73±14.78 | 36.56±9.1 | 24.98±5.75 | 0.13 | 0.13 |
| IL-12 (p70) | 213.23±161.57 | 36.26±15.2 | 89.86±48.31 | 48.21±18.29 | 0.49 | 0.63 |
| IL-13 | 280.52±113.66 | 230.23±100.22 | 133.25±24.27 | 85.05±14.86 | 0.25 | 0.21 |
| IL-15 | 561.24±317.01 | 187.52±27.11 | 176.42±23.84 | 175.85±51.45 | 0.27 | 0.85 |
| IL-17 | 48.82±8.38 | 5.35±1.57 | 122.81±38.54 | 11.09±3.39 | 0.11 | 0.17 |
| IP-10 | 335.58±139.1 | 80.37±31.76 | 201.89±73.21 | 154.79±32.85 | 0.42 | 0.13 |
| KC | 14566.26±3022.17 | 5346.43±1691.25 | 15509.23±3086.91 | 5054.74±2263.29 | 0.83 | 0.92 |
| LIF | 8.7±1.72 | 3.05±0.42 | 11.44±2.8 | 8.99±3.87 | 0.42 | 0.19 |
| LIX | 3601.52±1268.09 | 2085.57±1389.88 | 5746.47±2062.01 | 5858.41±729.93 | 0.40 | 0.04* |
| MCP-1 | 311.39±60.84 | 79.83±27.77 | 587.28±153.63 | 143.33±44.41 | 0.13 | 0.26 |
| M-CSF | 1713.43±1414.52 | 126.95±63.6 | 49.31±13.9 | 77.34±21.85 | 0.28 | 0.49 |
| MIG | 54.06±9.56 | 31.35±13.7 | 35.22±7.34 | 35.46±12.97 | 0.15 | 0.83 |
| MIP-1a | 338.62±29.83 | 387.17±116.48 | 303.02±60.38 | 284.9±70.6 | 0.61 | 0.47 |
| MIP-1B | 767.33±149.49 | 182.56±21.49 | 569.21±118.96 | 206.9±76.32 | 0.32 | 0.77 |
| MIP-2 | 956.25±268.55 | 235.63±41.12 | 722.63±212.49 | 224.02±36.02 | 0.51 | 0.84 |
| RANTES | 171.24±81.84 | 59.46±21.46 | 41.09±5.28 | 77.41±32.4 | 0.16 | 0.66 |
| TNFa | 46.26±12.49 | 30.15±6.14 | 52.64±17.94 | 50.78±13.13 | 0.78 | 0.20 |
| VEGF | 2.67±1.38 | 0.86±0.09 | 1.7±0.67 | 1.16±0.23 | 0.54 | 0.26 |

**Supporting Information Table S2**

**Major Resources Tables**

**Mouse Breeding:** Albumin-Cre mice in C57BL/6 background were from the Jackson Laboratory (stock NO: 003574). SR-BI ^fl/fl^ mice in 8x C57BL/6J background were from Dr. Chieko Mineo (University of Texas Southwestern Medical Center) and continued to 2x backcross to C57BL/6J.

|  | Vendor or Source | Breeding Strategy | Background Strain |
| --- | --- | --- | --- |
| Parent - Male | In house | Albumin-Cre mice | 10x C57BL/6J |
| Parent - Female | In house | SR-BI ^fl/fl^ mice | 10x C57BL/6J |

**Mouse Models (in vivo studies)**

| Mouse Model | Vendor or Source | Sex |
| --- | --- | --- |
| AlbCreSR-BI ^fl/fl^ mice | In house breeding | male & female |
| SR-BI ^fl/fl^ mice |  |  |
| LDLR-/- mice |  |  |
| C57BL/6J |  |  |

**Mouse Housing Conditions**

|  | Mouse Housing Conditions | Note |
| --- | --- | --- |
| Set Temperature range | 22 °C |  |
| Set Humidity range | 50% |  |
| Light Cycle (Mouse) | 14 hours: 10 hours |  |
| Water | RO Water | ad libitum |
| Standard Feed | Teklad Irradiated Global 18% Protein Rodent Diet (Envigo); Diet # 2918 | ad libitum |
| Standard Bedding | P.J. Murphy Coarse SaniChip |  |
| Experimental Feed | Teklad Irradiated Global 18% Protein Rodent Diet (Envigo); Diet # 2918 | ad libitum |
| SPF | Yes |  |

**Antibodies**

| **Antibody** | **Vendor or Source** | | **Catalog #** |
| --- | --- | --- | --- |
| **anti-SRBI** | Sigma | | customer made |
| **anti-ß-actin** | Sigma | | A5441 |
| **anti-mouse CD16/CD32 (Fc)** | Biolegend | | 101302 |
| **Percp-cy5.5-conjugated anti-CD11b (M1/70)** | BD Bioscience | | 550993 |
| **APC-conjugated anti-CD45 (30F11)** | Biolegend | | 103124 |
| **FITC-conjugated anti-Ly6C (HK1.4)** | Biolegend | | 128006 |
| **Reagents/Kits/Commercial Service** |  | |  |
| **Reagents** | | **Vendor or Source** | **Catalog #** |
| **Free Cholesterol E** | | Wako | 435-35801 |
| **Total Cholesterol E** | | Wako | 439-17501 |
| **Hemoglobin assay kit** | | Sigma | MAK 115-1KT |
| **Cytokine panel assay** | | EVE Technologies |  |
| **30% Acrylamide/Bis solution** | | Bio-rad | 161-0156 |
| **APS** | | Bio-rad | 161-0700 |
| **Resolving gel buffer 1.5M Tris-HCL PH 8.8** | | Bio-rad | 161-0798 |
| **Stacking gel buffer 0.5M Tris-HCL PH 6.8** | | Bio-rad | 161-0799 |
| **TEMED** | | Bio-rad | 161-0800 |
| **ECL** | | Fisher | 32106 |
| **#23G needle** | | Fisher | 14-826A |
| **Probucol** | | Sigma | P9672-50G |
| **Red taq** | | Bioline | bio-25044 |
| **Proteinase K** | | Invitrogen | 25530-031 |
| **Agrose** | | Invitrogen | 16500-500 |
| **DPBS** | | Sigma | D8662-500 |
| **Fatty acid free BSA** | | Sigma | A7030-100g |
| **Reticulocyte Stain** | | Sigma | R4132-120ML |
| **RNeasy Mini Kit** | | QIAGEN | 74104 |

**Primers**

| **Primer** | **Sequence 5' --> 3'** | **Primer Type** | **Vendor or Source** |
| --- | --- | --- | --- |
| **20239** | TGC AAA CAT CAC ATG CAC AC | Wild type Forward | Integrated DNA Technologies IDT |
| **20240** | TTG GCC CCT TAC CAT AAC TG | Common | Integrated DNA Technologies IDT |
| **oIMR5374** | GAA GCA GAA GCT TAG GAA GAT GG | Mutant Forward | Integrated DNA Technologies IDT |
